# High-Shear Granulation of Hygroscopic Probiotic-Encapsulated Skim Milk Powder: Effects of Moisture-Activation and Resistant Maltodextrin

**DOI:** 10.3390/ph16020217

**Published:** 2023-01-31

**Authors:** Andres Letona, Sungahm Ahn, Suyeon An, Daebeom Yun, Young-Rok Kim, Mario Muralles, Donghwa Chung

**Affiliations:** 1 Institute of Food Industrialization, Institutes of Green Bio Science and Technology, Seoul National University, Pyeongchang 25354, Republic of Korea; 2 Food Technology Major, Graduate School of International Agricultural Technology, Seoul National University, Pyeongchang 25354, Republic of Korea; 3 Department of Food Science and Biotechnology, Institute of Life Science and Resources, Kyung Hee University, Yongin 17104, Republic of Korea; 4 School of Materials Science and Engineering, Nanyang Technological University, 50 Nanyang Avenue, Singapore 639798, Singapore; 5 Center for Food and Bioconvergence, Seoul National University, Seoul 08826, Republic of Korea

**Keywords:** high-shear granulation, moisture-activation, resistant maltodextrin, probiotics, moisture sorption isotherm, flowability

## Abstract

A fine, hygroscopic, and poorly flowable probiotic powder encapsulating *Lactobacillus rhamnosus* GG (LGG) was granulated using a high-shear granulation process, wherein a small amount of water (4%, *w/w*) was used for moisture-activation with or without 10% (*w/w*) resistant maltodextrin (RM). The process consisted of four steps; premixing, agglomeration, moisture absorption, and drying steps. The moisture content, water activity, and viable cell count were monitored during the granulation. The size, morphology, and flowability of the granules were determined. The powder was successfully converted to about 10-times-larger granules (mass mean diameter = 162–204 µm) by this process, and the granules had a ‘snowball’ morphology. The LGG cells were well preserved under the high-shear granulation conditions, and the viable cell count of the granules greatly exceeded the minimum therapeutic level recommended for probiotic powders. The addition of RM decreased the moisture content of the granules; improved cell resistance to drying stress; narrowed the particle size distribution, with reductions seen in both very fine and very large particles; and produced more flowable granules. Moisture sorption analysis and differential scanning calorimetry demonstrated that these positive effects of RM on granulation were primarily attributed to its water distribution ability rather than its glass transition-related binding ability.

## 1. Introduction

Spray drying has been used extensively for several decades in the production of food powders. A major limitation of this process is that the powder has a small particle size (often <50 µm in mean diameter), which causes dust-like behavior and creates significant difficulties in terms of handling, processing, and storage [1,2,3,4,5,6]. An effective way to solve such limitations is to transform the fine spray-dried powders into larger agglomerated granules [7,8].

High-shear wet granulation (HSWG) is a widely used granulation technique in pharmaceutical, cosmetic, and food industries, wherein the wetting/nucleation, consolidation/growth, and attrition/breakage of particles occur during the injection of an aqueous solution under the strong shear force generated by an impeller [9,10]. This granulation technique is easy to control and scale up and has a short process time [11]. However, HSWG often uses large amounts of aqueous solution, resulting in over-wetting and over-granulation, especially in the granulation of moisture-sensitive hygroscopic powders; this can lead to undesirable wall adhesion and big lumps or granules [12,13,14]. Therefore, additional steps for drying, using tray or fluidized-bed dryers, and for crushing large lumps and granules are often required for HSWG.

To reduce such limitations of HSWG, a modified process called moisture-activated dry granulation (MADG) has been introduced. This process forms granules via the same mechanism as HSWG but differs from HSWG in the following points: in the first step, called the “agglomeration step”, MADG uses a relatively small amount of aqueous solution (about 1–4%), and, in the second step, called the “moisture absorption step”, a moisture absorbent, such as microcrystalline cellulose, is often added to absorb and redistribute excess water. Consequently, the MADG process does not typically require an additional drying step [13,14,15,16,17,18]. Compared with the typical HSWG, MADG produces relatively small granules with a narrow size distribution, good flowability, and reduced segregation [19,20,21]. However, even with MADG, serious over-wetting and over-granulation are expected to occur, especially during the granulation of fine (large surface area) and highly hygroscopic powders, due to the failure of the homogeneous distribution of sprayed water.

In this study, a fine, hygroscopic, and poorly flowable probiotic powder, which was produced by spray-drying reconstituted skim milk (RSM) fermented with *Lactobacillus rhamnosus* GG (LGG), was granulated by a four-step high-shear granulation process based on the MADG concept, i.e., premixing, agglomeration, moisture absorption, and drying steps. In this granulation process, lactose monohydrate and microcrystalline cellulose were used as a filler and moisture absorbent, respectively, as in other typical MADG or tableting processes [13,14,15,16,17,18]. In our preliminary experiments, it was found that this highly hygroscopic spray-dried probiotic powder was not successfully granulated by typical HSWG or MADG, and serious over-wetting and over-granulation were observed. In this study, therefore, a portion of microcrystalline cellulose was added in the premixing step to reduce over-wetting and the remaining microcrystalline cellulose was added in the moisture absorption step. Also, a short drying step was added to reduce over-granulation after particle agglomeration. In addition, resistant maltodextrin (RM; a resistant starch type V) was used to mitigate over-wetting and over-granulation. RM is an amorphous fiber and was thus expected to have higher water absorption ability than microcrystalline cellulose or lactose monohydrate (crystalline form) and thereby to redistribute localized excess water more effectively during the granulation process. Furthermore, RM, like common maltodextrin, may protect probiotic cells during the granulation process, when high-shear and heat treatments are involved, and functions as a prebiotic and dietary fiber [22]. Hence, the use of RM with probiotic powder is worth exploring as a method for formulating synbiotic products. The moisture content, water activity, and viable cell count were monitored in every stage of the granulation process. Granule properties, including granule size distribution, morphology, and flowability, were determined.

## 2. Results and Discussion

### 2.1. High-Shear Granulation of SD Powder

**Figure 1:**
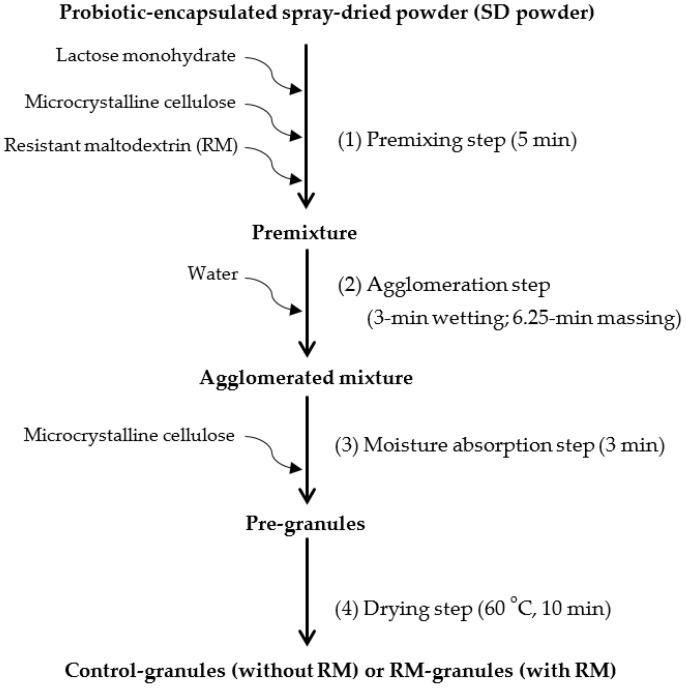
Experimental scheme of high-shear granulation process performed in the study.

The SD powder, which is a fine, highly hygroscopic, and poorly flowable probiotic powder produced by spray-drying RSM fermented with LGG, was successfully granulated by the high-shear granulation process used in this study, regardless of the addition of RM in the premixing step (Figure 1). This process used moisture-activation for particle agglomeration, achieved by spraying a small amount of water (4%, *w/w*) as in MADG but differing from the original MADG process proposed by Ullah et al. [13] in that 70% (*w/w*) of microcrystalline cellulose (moisture absorbent) was added in the premixing step to avoid the over-wetting and over-agglomeration of the SD powder; the remaining 30% was added in the moisture absorption step. In addition, a short drying step (60 °C for 10 min) was added after the agglomeration step to reduce over-granulation and to control the final moisture content (*M*) and water activity (*a*_w_) of the granules, which is often omitted in MADG. No significant wall adhesion or large lump formation was observed during the formation of pre-granules in the granulator. To better understand this granulation process, the cell viability, *M*, and *a*_w_ were monitored in each step of the process.

### 2.2. Moisture Content, Water Activity, and Viable Cells during High-Shear Granulation

**Figure 2:**
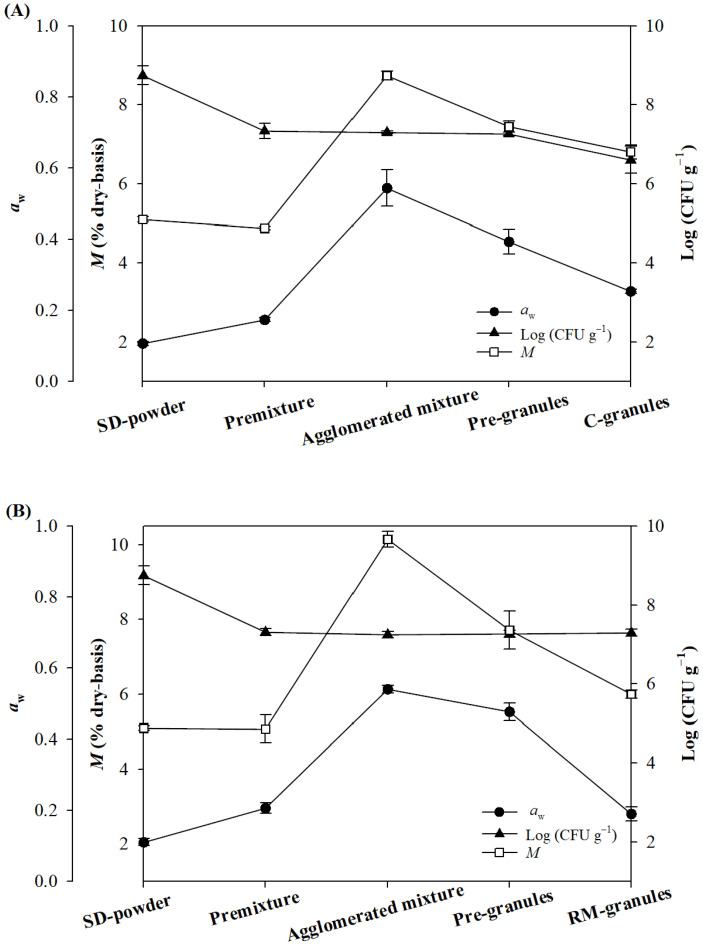
Changes in moisture content (*M*), water activity (*a*_w_), and viable cells during high-shear granulation process conducted for the production of (**A**) Control-granules and (**B**) RM-granules.

**Figure 3:**
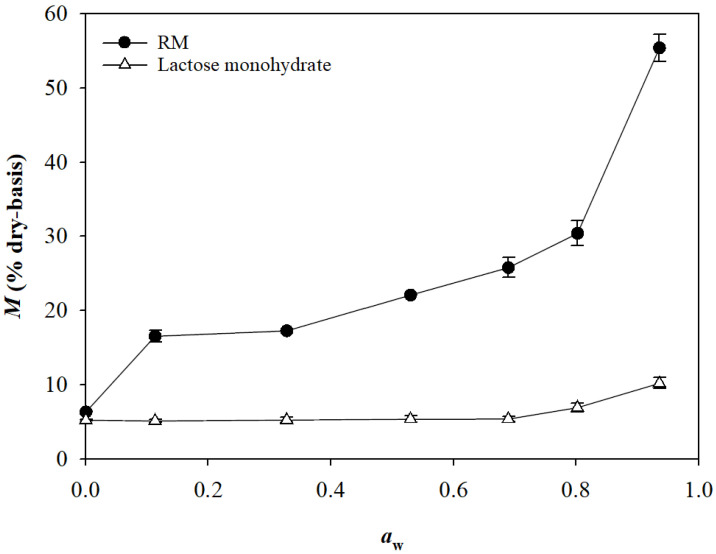
Moisture sorption isotherms of lactose monohydrate and resistant maltodextrin (RM) at 25 °C.

Figure 2 presents the *M*, *a*_w_, and viable cell count, measured after the premixing, agglomeration, moisture absorption, and drying steps of the two high-shear granulation processes used in this study for the production of Control-granules (without RM) and RM-granules (with RM). All parameter values are summarized in Table S1. In the high-shear granulation process for the Control-granules, *M* increased from 5.10% to 8.81% after the agglomeration step due to the water spraying, decreased to 7.53% after the moisture absorption step due to the addition of microcrystalline cellulose, and then further decreased to 6.86% after the drying step. In the granulation process for the RM-granules, a more significant increase in *M* was observed after the agglomeration step (from 5.10% to 10.15%), although the final *M* value (6.00%) of the RM-granules was even smaller than that of the Control-granules. The steeper increase in *M* during the agglomeration step of the RM-granule preparation process compared to the granulation process for the Control-granules occurred because RM was much more hygroscopic than lactose monohydrate. Figure 3 shows that RM had a typical S-shaped Type II moisture sorption isotherm (MSI) curve, as reported for other conventional and resistant maltodextrin [23,24], while lactose monohydrate exhibited a J-shaped MSI curve, which is typically observed for crystalline sugars [25]. Comparison of the MSIs revealed that RM was much more hygroscopic than lactose monohydrate and that RM showed an *M* value about four times higher than lactose monohydrate when equilibrated at an *a*_w_ of 0.53, which was close to the *a*_w_ of the mixtures (0.54) measured after the agglomeration step. The *a*_w_ of both pre-granular mixtures significantly increased from 0.11 to 0.54 during the agglomeration step and then decreased to 0.25 and 0.19 when the Control- and RM-granules were formed at the end of the process, respectively, in accordance with the change in *M* (Figure 2; Table S1). RM-granules showed lower values of *M* (6.00%) and *a*_w_ (0.19) than Control-granules (*M* = 6.86%, *a*_w_ = 0.25) (Table S1) despite the more hygroscopic nature of RM; this was probably because the RM granules had a smaller average size and more homogeneous size distribution (see Section 2.3), resulting in more effective drying at the final stage of the process.

The viable cell count, measured as 8.75 log (CFU g^−1^) for the SD powder, showed a sudden decrease after the premixing step due to the dilution caused by mixing with other ingredients in both granulation processes (Figure 2; Table S1). In the granulation process for the Control-granules, the viable cell count did not significantly change during the agglomeration and moisture absorption steps but slightly decreased to 6.60 log (CFU g^−1^) after the drying step. In the process for the RM-granules, the viable cell count remained almost the same after the agglomeration step and even after the drying step, yielding a cell count of 7.29 log (CFU g^−1^) at the end of the process. The viable cell counts of both granules met the minimum therapeutic level (6 log (CFU g^−1^)) recommended for probiotic powders [26,27,28,29]. The viable cell count of the RM-granules remained almost the same after storage at 25 °C for 28 days, while that of the Control-granules slightly decreased (Table S2). The results indicate that the high shear force generated by the impeller and chopper of the granulator did not significantly influence cell viability due to the protective effect of encapsulation within the protein-based matrix structure of the SD powder. The results also demonstrate that RM provided probiotic cells with resistance to drying stress.

### 2.3. Morphology, Crystallinity, and Particle Size

**Figure 4:**
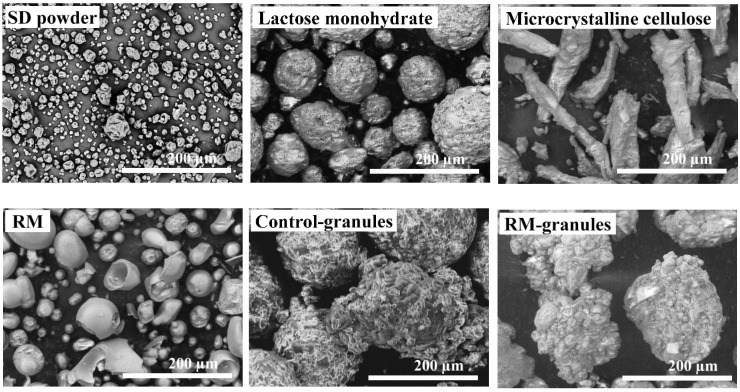
SEM images of SD powder, lactose monohydrate, microcrystalline cellulose, resistant maltodextrin (RM), Control-granules, and RM-granules.

Figure 4 presents scanning electron microscopy (SEM) images of the ingredients used in the high-shear granulation process and the two types of granules produced. The SD powder and lactose monohydrate and RM particles displayed a semispherical shape, with volume-weighted mean diameters (*d*_4,3_) of 14.51, 110.25, and 80.67 µm, respectively, while microcrystalline cellulose particles had a rod-type shape with a *d*_4,3_ of 118.70 µm (Figure 4; Table S3). The Control- and RM-granules displayed a ‘snowball’ morphology due to the consolidation of ingredients and growth of consolidated particles, which is typically observed for granules produced by the HSWG process [5]. No significant difference in morphology was visually observed between the two types of granules.

The crystalline structure of each ingredient and the two types of granules produced was examined by X-ray diffraction (XRD) analysis (Figure S1). Lactose monohydrate showed seven characteristic crystalline peaks at a diffraction angle (2*θ*) of 12.5°, 16.4°, 19.1°, 19.6°, 20.0°, 21.2°, and 23.7°, while microcrystalline cellulose exhibited two broad peaks at a 2*θ* of 15.2° and 22.5°, indicating that it is an amorphous, glassy, or disordered nanocrystalline material [30]. RM and SD powder showed halo-shaped XRD patterns without any characteristic peaks (Figure S1), indicating that these ingredients had completely amorphous structures. The Control- and RM-granules showed almost the same XRD patterns as lactose monohydrate, although the peak intensities were smaller than those of lactose monohydrate (Figure S1). This implies that, during the high-shear granulation process, lactose monohydrate maintained the crystalline structure and that no other crystallization reactions occurred.

**Figure 5:**
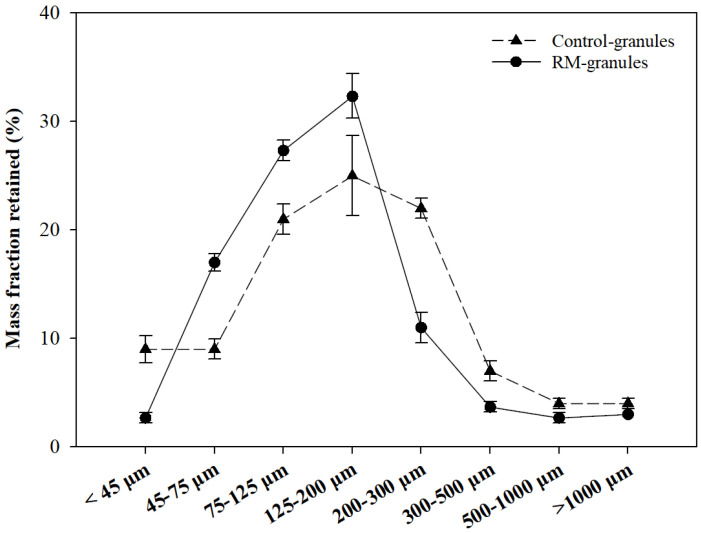
Particle size distribution of Control- and RM-granules based on mass fraction obtained by sieve analysis.

Figure 5 presents the particle size distribution of the Control- and RM-granules based on the mass fraction obtained by sieve analysis. For the Control-granules, about 77% of the granules lay between 75 and 200 µm, and the highest fraction (about 25%) was observed in the size range of 125–200 µm. On the other hand, for the RM-granules, about 87% of the granules lay between 75 and 200 µm, and the highest fraction (about 32%) was observed in the size range of 125 to 200 µm. The RM-granules showed a larger *d*_10_ (57.67 µm), a much smaller *d*_90_ (294.67 µm), and a smaller span value (1.18) compared with the Control-granules (*d*_10_ = 34.33 µm, *d*_90_ = 436.67 µm, and span = 2.48) (Table 1). In particular, the mass fraction of very fine particles (<45 µm), which often cause problems in particle handling, was much smaller in the RM-granules (3%) than Control-granules (9%). The results indicate that the RM-granules had a narrower size distribution compared to the Control-granules, suggesting that the addition of RM to the formulation improved the distribution of water during the agglomeration and moisture absorption steps, resulting in a reduction of very fine particles and very large agglomerates. The mass mean diameters (*d*_m_) of the Control- and RM-granules were 204.20 µm and 161.98 µm, respectively (Table 1), indicating that the size of SD powder increased about 10-fold during this high-shear granulation process.

### 2.4. Density, Porosity, and Flowability

The true densities (*ρ*_true_) of the two types of granules (1.50–1.52 g cm^−3^) were larger than that of the SD powder (1.46 g cm^−3^) (Table 2), indicating that the SD powder was well agglomerated during the high-shear granulation such that the two types of granules had greater structural integrity than the SD powder. The two types of granules showed significantly lower values of loose bulk density (*ρ*_lb_ = 0.48–0.52 g cm^−3^) and tapped bulk density (*ρ*_tb_ = 0.57–0.58 g cm^−3^) compared with the SD power (*ρ*_lb_ = 0.54 g cm^−3^, *ρ*_tb_ = 0.73 g cm^−3^) (Table 2). This is because the two types of granules had a larger void volume (*ε* = 61.2–62.7%) than the SD powder (*ε* = 50.1%) due to their larger particle sizes. Larger particles tend to be less tightly packed compared with smaller ones, thus yielding a larger *ε* value [31,32].

The Carr compressibility index (CI) and Hausner ratio (HR) of the SD powder were 26.02% and 1.35, respectively (Table 2), indicating that the powder had ‘poor’ flowability according to the criteria published by the US Pharmacopeia (USP) (Table S4) [33]. The CI and HR values were greatly reduced, to 14.40% and 1.17, respectively, after the high-shear granulation, thereby forming the Control-granules (Table 2), which showed ‘good’ flowability according to the USP criteria. The improvement of particle flowability achieved by the high-shear granulation was primarily attributed to an enlargement of particle size. The increased particle size caused a reduction in particle surface area, which diminished not only the friction between particles but also interparticle cohesion by van der Walls attractions [34]. The RM-granules showed smaller values of CI (10.79%) and HR (1.12) than the Control-granules (Table 2). This indicated that the RM-granules had better flowability than the Control-granules, although the two types of granules belonged to the same ‘good’ class according to the USP criteria. The better flowability of the RM-granules may be due to their narrower size distribution (Figure 5) and, especially, the smaller mass fraction of very fine particles (Figure 5, Table 1), which can greatly increase friction and van der Waals cohesion between particles.

### 2.5. Role of RM in High-Shear Granulation

As discussed in previous Sections, the replacement of 22% of lactose monohydrate by RM provided several interesting benefits during the production of granules by high-shear granulation, i.e., a lower final *M* value, increased cell resistance to drying stress, narrower particle size distribution, reduction in the fraction of very fine and large particles, and better flowability. These benefits were primarily attributed to the higher water absorption of the amorphous RM compared with the crystalline lactose monohydrate. Accordingly, RM could redistribute localized excess water more effectively during agglomeration and moisture absorption, which reduced over-wetting and over-agglomeration. In addition, RM conferred heat stability on probiotic cells like common maltodextrin.

**Figure 6:**
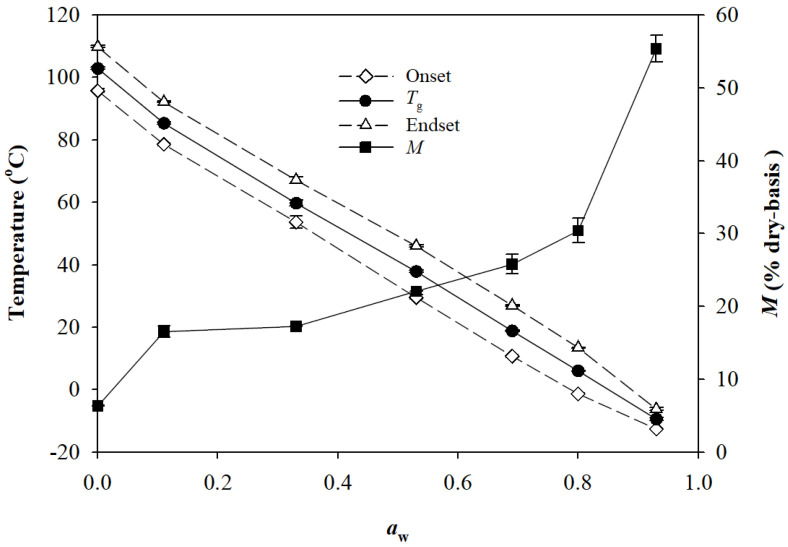
Onset, mid, and endset temperatures of endothermic steps in DSC thermograms of resistant maltodextrin (RM), equilibrated at 25 °C under different relative humidities (RH). The RH was expressed as water activity (*a*_w_ = RH/100), and the midpoint was used as glass transition temperature (*T*_g_).

Considering its effects on particle size distribution, it was expected that RM could also act as a granulation binder to facilitate particle agglomeration. To explore the role of RM as a granulation binder, the glass transition of RM was investigated, because PVP K12 (3500 Da), a synthetic polymeric granulation binder widely used in pharmaceutical products, is known to undergo a glass-to-rubbery transition for its binding activity upon spraying water during the agglomeration stage [35,36]. Figure 6 presents the values of glass transition temperature (*T*_g_; including the onset and endset temperatures) and *M* of RM, equilibrated at 25 °C under different *a*_w_ conditions. The *T*_g_ of RM rapidly decreased from 102.9 °C to 37.9 °C with increasing *a*_w_ from 0 to 0.53 and further decreased to −9.4 °C when *a*_w_ reached 0.94, due to the plasticizing effect of water. During the granulation process of the RM-granules, *a*_w_ increased to 0.54 after the agglomeration step (Figure 2; Table S1). Considering that RM had a *T*_g_ of 37.9 °C at *a*_w_ = 0.53 and that the agglomeration step proceeded at room temperature (24 ± 1 °C), RM in the premixture existed in the glassy state during agglomeration. During the drying step, the temperature was raised to 60 °C, but *a*_w_ rapidly decreased to 0.19, at which the *T*_g_ of RM was about 75 °C. Therefore, RM in the agglomerated mixture mostly existed in the glassy state during drying. The results indicate that the beneficial effect of RM on particle size distribution was not due to its binding ability (related to the glassy-to-rubber transition) but rather to its highly hygroscopic nature. Figure 6 shows that the glass-to-rubber transition of RM was initiated at 24 °C when *a*_w_ reached about 0.58. Therefore, it is suggested that RM can be used as an effective natural prebiotic binder in granulation processes wherein a higher amount of water is used, such as the HSWG process, rather than in the granulation process in this study or the common MADG process wherein only a small amount of water is used for moisture-activation.

## 3. Materials and Methods

### 3.1. Materials

LGG (ATCC 53103) was purchased from the American Type Culture Collection (Manassas, VA, USA). Skim milk powder (SMP; 52% (*w/w*) carbohydrate, 49% (*w/w*) sugars, 37% (*w/w*) protein, 0.5% (*w/w*) lipid, 0.54% (*w/w*) sodium, and 1% (*w/w*) calcium) was obtained from Seoul Milk Co., Ltd. (Seoul, Korea). De Man, Rogosa, and Sharpe (MRS) broth and yeast extract were purchased from Difco Laboratories Inc. (Detroit, MI, USA), and glucose was purchased from Ducksan Pure Chemicals Co., Ltd. (Asan, Korea). Phosphorous pentoxide (P_4_O_10_) was obtained from Daejung Co., Ltd. (Shiheung, Korea). Lithium chloride (LiCl), magnesium chloride (MgCl_2_), and magnesium nitrate (Mg(NO_3_)_2_) were purchased from Samchun Pure Chemical Co., Ltd. (Seoul, Korea). Potassium iodide (KI), ammonium sulfate ((NH_4_)_2_SO_4_), and potassium nitrate (KNO_3_) were obtained from Duksan Pure Chemical Co., Ltd. Microcrystalline cellulose (Avicel^®^ PH-102), α-lactose monohydrate (Flowlac^®^ 100), and RM (Fibersol-2^®^; 1800 Da) were purchased from Pharmaline (Suwon, Korea), MEGGLE Group (Wasserburg, Germany), and Matsutani Korea (Seongnam, Korea), respectively.

### 3.2. Preparation of LGG-Containing Spray-Dried Powder

The LGG-containing feed suspension for spray drying was prepared according to Lim et al. [1]. A medium (190 mL) of RSM was prepared by dissolving SMP, glucose, and yeast extract in distilled water at 10% (*w/w*), 2% (*w/w*), and 1% (*w/w*), respectively. LGG was subcultured at 37 °C in MRS broth (20 mL), and the subculture was inoculated into the RSM medium at 5% (*w/w*). The RSM medium was incubated at 42 °C in a water bath with stirring at 100 rpm until the pH decreased from 6.6 to 3.9 (~9.2 log (CFU g^−1^)). SMP was further added to the LGG-fermented RSM to a final concentration of 30% (*w/w*) with stirring for 30 min to prepare the feed suspension for spray drying. The feed suspension was spray-dried using a laboratory-scale spray dryer equipped with a single nozzle (0.7 mm diameter) and concurrent airflow system (Eyela SD-1000; Tokyo Rikakikai Co. Ltd., Tokyo, Japan). The spray drying was performed at an inlet temperature of 160 ± 1 °C, outlet temperature of 80 ± 1 °C, feed flow rate of 300 mL h^−1^, atomization pressure of 100 kPa, and hot air flow rate of 0.20−0.24 m^3^ min^−1^. The spray-dried powder (SD powder) was cooled to room temperature, collected from the dryer, and stored at room temperature in a hermetically sealed jar.

### 3.3. High-Shear Granulation with Moisture-Activation

The SD powder was granulated using a high-shear granulator (NMG-1 L; Nara Machinery Co., Tokyo, Japan), equipped with a bowl (1 L) and air brush (0.5 mm nozzle diameter, 0.5 bar atomizing air pressure; BBA-M001; Yamato Korea Co., Seoul, Korea). The granulation was performed according to Ullah et al. [13,14,15] with some modifications (Figure 1). The granulation process consisted of four steps; premixing, agglomeration, moisture absorption, and drying. In the premixing step, the SD powder was mixed with lactose monohydrate (filler) and microcrystalline cellulose (moisture absorbent) for 5 min in the granulator bowl. To investigate the effects of RM on granulation, 22% (*w/w*) of lactose monohydrate was replaced by RM. In the agglomeration step, distilled water was sprayed over the premixture periodically so that the wetting and massing of the premixture could be repeated during the granulation. Wetting by spraying for 12 s and subsequent massing for 25 s without spraying were repeated 15 times. The total wetting and massing times were 3 and 6.25 min, respectively. The total amount of water sprayed was 4% (*w/w*). This periodic spraying allowed the sprayed water to be more effectively distributed within the premixture and reduced powder adhesion on the wall and bottom of the granulator. In the moisture absorption step, microcrystalline cellulose was further added and mixed for 3 min. In the drying step, the formed granules were heated in an oven at 60 °C for 10 min. The total mass of the ingredients used in the granulation process was adjusted to 200 g. High-shear was created using an impeller operating at 300 rpm and a chopper operating at 1000 rpm. The granules produced without RM were designated as ‘Control-granules’ and those formed in the presence of RM were designated as ‘RM-granules’. Both types of granules were stored at room temperature in airtight aluminum bags. The compositions of the granules are summarized in Table 3.

### 3.4. Microstructure

The microstructure of granules was examined using SEM (TM3030 Plus; Hitachi, Tokyo, Japan). The obtained granules were mounted on an aluminum sample mount using double-sided adhesive tape, and the micrographs (500×) were acquired at 15 kV.

### 3.5. X-ray Diffraction Analysis

The XRD patterns of each ingredient and Control- and RM-granules were obtained using a SmartLab X-ray diffractometer (Rigaku, Tokyo, Japan). All samples were equilibrated under the condition of 25 °C and zero RH in desiccators containing saturated P_4_O_10_ solution before analysis. Each sample was placed in a sample holder (1.5 cm × 1.5 cm × 0.2 cm), and CuKα radiation (λ = 1.54 Å) was used to obtain diffractograms at 40 kV and 40 mA (step size = 0.02°/s and diffraction angle (2*θ*) = 5–50°). The peak intensity was expressed as counts per second (cps) and plotted with respect to 2*θ*.

### 3.6. Particle Size Analysis

The *d*_4,3_ (µm) of the SD powder was determined using a laser diffraction particle size analyzer (1190LD; CILAS, Orleans, France). The particle size distribution of the Control- and RM-granules were obtained by sieve analysis performed using a sieve shaker (Minor 200; Endecotts, London, UK) equipped with sieves (200 mm diameter) of different mesh sizes (45–1000 μm). Granules (200 g) were sieved for 10 min with a continuous vibration speed of 3000 min^−1^ at 50 Hz. The *d*_m_ of the granules was determined according to the following equation:

(1) $$d_{m}=\frac{\sum_{i=1}^{n}m_{i}{\bar{d}}_{i}}{\sum_{i=1}^{n}m_{i}}\times 100$$

where *m*_i_ is the mass of granules in a sieve of mesh size from *d*_i_ to *d*_i+1_, and $\bar{d_{i}}$ is the arithmetic mean of *d*_i_ and *d*_i+1_. The values of *d*_10_, *d*_50_, and *d*_90_ were also determined; these represent particle diameters below which the mass percentage of particles is 10%, 50%, and 90%, respectively, in the cumulative particle size distribution. The span value, representing the uniformity of the particle size distribution, was also calculated.

### 3.7. Determination of Density, Porosity, and Flowability

The *ρ*_true_ of the SD powder and Control- and RM-granules was determined using a gas pycnometer (Ultrapyc 1200e; Quantachrome Instruments, Boynton Beach, FL, USA). The *ρ*_lb_ was determined by measuring the volume of the particles (10 g) in a 50 mL graduated cylinder. The *ρ*_tb_ was obtained by measuring the particle volume after manually tapping the cylinder 100 times. The *ε* of the particles was determined as follows:

(2) $$\varepsilon =\frac{\left(\rho_{\mathrm{true}}-\rho_{\mathrm{tb}}\right)}{\rho_{\mathrm{true}}}\times 100$$

The flowability of the particles was determined according to the values of CI and HR (Table S4), calculated as follows:

(3) $$\mathrm{CI}=\frac{\left(\rho_{\mathrm{tb}}-\rho_{\mathrm{lb}}\right)}{\rho_{\mathrm{tb}}}\times 100$$

(4) $$\mathrm{HR}=\frac{\rho_{\mathrm{tb}}}{\rho_{\mathrm{lb}}}$$

### 3.8. Moisture Content and Water Activity Measurements

The *M* (% dry-basis) of the SD powder and two types of granules were determined gravimetrically, wherein the weight loss of the samples was measured after oven-drying at 105 °C for 24 h [37]. The *a*_w_ of the particles was determined at 25 °C using a digital water activity meter (Series 3 TE; Aqualab, Decagon, WA, USA).

### 3.9. Determination of Viable Cells

The viable cell counts of LGG in the SD powder and two types of granules were determined using the plate count method according to Lim et al. [1]. Particles (1 g) were dispersed in a sterile saline solution of 0.85% NaCl (9 mL) and vortexed for 1 min, followed by decimal dilution. Aliquots of the dilutions were plated on sterile MRS agar, followed by incubation at 37 °C for 48 h. The viable cells were counted and expressed as log CFU g^−1^.

### 3.10. Determination of Moisture Sorption Isotherms of Lactose Monohydrate and RM

The MSIs of the lactose monohydrate and RM powders were determined at 25 °C using the gravimetric method of Yu et al. [38]. Briefly, lactose monohydrate or RM powder samples (~2 g) were spread evenly on pre-weighed aluminum dishes and placed in desiccators containing saturated P_4_O_10_ solution (0% relative humidity (RH)), followed by incubation at 25 °C until the mass change was <0.01 g. The samples were then transferred to desiccators containing saturated salt solutions of LiCl (11% RH), MgCl_2_ (33% RH), Mg(NO_3_)_2_ (53% RH), KI (69% RH), (NH_4_)_2_SO_4_ (80% RH), or KNO_3_ (94% RH) and further incubated at 25 °C until the mass change was <0.01 g. The *M* of the samples at equilibrium were measured by oven-drying at 105 °C for 24 h [37] and plotted with respect to water activity (*a*_w_ = RH/100) to obtain MSIs.

### 3.11. Determination of the Glass Transition of RM

The *T*_g_ of RM was determined with respect to *a*_w_ using a differential scanning calorimeter (DSC) (DSC 250; TA instruments, New Castle, DE, USA). A sample of RM (~5 mg), prepared for the determination of MSI as described in Section 3.9, was placed in an aluminum pan and hermetically sealed with a lid. The DSC was run twice from −50 °C to 150 °C at a scanning rate of 10 °C min^−1^ with nitrogen purging at a flow rate of 50 mL min^−1^. The onset, mid, and endset temperatures of the endothermic steps in DSC thermograms were determined using TRIOS software (TA Instruments), and the midpoint was used as *T*_g_.

### 3.12. Statistical Analysis

All measurements were performed at least in triplicate. Data are presented as mean ± standard deviation. The means were compared by one-way analysis of variance (ANOVA) and Tuckey’s test, with *p* ≤ 0.05 taken to indicate a significant difference. Data analysis was carried out using IBM SPSS Statistics 26.0 software (IBM Co., Armonk, NY, USA).

## 4. Conclusions

This study successfully granulated LGG-encapsulating SD powder, which was fine, highly hygroscopic, and poorly flowable, via a four-step high-shear granulation process. This process used moisture-activation with a small amount of water (4%, *w/w*) and microcrystalline cellulose but differed from the original MADG process in that microcrystalline cellulose was added not only in the moisture absorption step but also in the premixing step to avoid over-agglomeration during the agglomeration step. Furthermore, a short drying step was added after the agglomeration step to reduce over-granulation and to control the final moisture of the granules. The Control- and RM-granules showed a similar ‘snowball’ morphology, which is typically observed for particles formed by consolidation. This granulation process increased the fine SD powder size about 10-fold, and most of the granules were in the size range of 125 to 200 µm. The LGG cells were well preserved under high-shear conditions due to the protective effect of the protein-based SD powder, and the viable cell counts of both Control- and RM-granules met the minimum therapeutic level (6 log (CFU g^−1^)) recommended for probiotic powders. The replacement of 22% of lactose monohydrate by RM provided several benefits. It decreased the granule moisture content, increased cell resistance to drying stress, narrowed the particle size distribution, reduced the fractions of both very fine and very large particles, and yielded better flowability. These beneficial effects were primarily attributed to the higher hygroscopicity of amorphous RM compared with crystalline lactose monohydrate, which allowed RM to more effectively redistribute localized excess water during the granulation process without significant over-wetting and over-agglomeration. It was expected that RM, unlike PVP, did not have a particle binding ability related to the glass-to-rubber transition. This study provides practical information on the use of moisture-activation and RM, a prebiotic dietary fiber, in the production of potential synbiotic products via a high-shear granulation process.

## Figures and Tables

**Table 1:** Diameters of Control- and RM-granules measured by sieve analysis.

Size Parameter	Control-Granules	RM-Granules
*d*_m_ (µm)	204.20 ± 7.65	161.98 ± 3.15
*d*_10_ (µm)	34.33 ± 2.05	57.67 ± 0.47
*d*_50_ (µm)	162.00 ± 3.56	131.33 ± 2.05
*d*_90_ (µm)	436.67 ± 15.33	294.67 ± 4.64
Span	2.48 ± 0.10	1.81 ± 0.05

*d*_m_ = mass mean diameter; *d*_10_, *d*_50_, and *d*_90_ = particle diameters below which the mass percentage of particles is 10%, 50%, and 90%, respectively, in the cumulative particle size distribution.

**Table 2:** True density (*ρ*_true_), loose bulk density (*ρ*_lb_), tapped bulk density (*ρ*_tb_), Carr compressibility index (CI), and Hausner ratio (HR) of SD powder, Control-granules, and RM granules.

	*ρ*_true_ (g cm^−3^)	*ρ*_lb_ (g cm^−3^)	*ρ*_tb_ (g cm^−3^)	*ε* (%)	CI (%)	HR
SD powder	1.46 ± 0.00 ^c^	0.54 ± 0.00 ^a^	0.73 ± 0.00 ^a^	50.1 ± 0.2 ^c^	26.02 ± 0.52 ^a^	1.35 ± 0.01 ^a^
Control-granules	1.52 ± 0.01 ^a^	0.48 ± 0.00 ^c^	0.57 ± 0.01 ^b^	62.7 ± 0.8 ^a^	14.40 ± 1.16 ^b^	1.17 ± 0.02 ^b^
RM-granules	1.50 ± 0.00 ^b^	0.52 ± 0.00 ^b^	0.58 ± 0.00 ^b^	61.2 ± 0.2 ^b^	10.79 ± 0.56 ^c^	1.12 ± 0.01 ^c^

SD powder = probiotic-encapsulated spray-dried powder; RM = resistant maltodextrin. Values with different letters in the same column are significantly different at *p* ≤ 0.05 according to Tukey’s test.

**Table 3:** Compositions of ingredients added during the high-shear granulation for the production of Control- and RM-granules.

Steps	Ingredients Added	Composition (%, *w/w*)	
		Control-Granules	RM-Granules
Premixing	SD powder	25.0	25.0
	Lactose monohydrate	46.0	36.0
	Microcrystalline cellulose	17.5	17.5
	Resistant maltodextrin (RM)	-	10.0
Agglomeration	Water	4.0	4.0
Moisture absorption	Microcrystalline cellulose	7.5	7.5
Total		100	100

SD powder = probiotic-encapsulated spray-dried powder.

## Data Availability

Data is contained within the article and the Supplementary Materials.

## Supplementary Materials

The following supporting information can be downloaded at: https://www.mdpi.com/article/10.3390/ph16020217/s1, Table S1: Moisture content (*M*), water activity (*a*_w_), and viable cells (log (CFU g^−1^)) during high-shear granulation process conducted for the production of Control- and RM-granules; Table S2: Viable cells (log (CFU g^−1^)) during the storage of Control- and RM-granules at 25 °C for 28 days; Table S3: Volume-weighted mean diameter (*d*_4,3_) of each ingredient used in high-shear granulation process; Table S4: Classification of powder flowability according to Carr compressibility index (CI) and Hausner ratio (HR); Figure S1: XRD diffractograms of each ingredient used in high-shear granulation process and Control- and RM-granules. The samples were equilibrated at 25 °C and zero RH before analysis.

## References

[1] Lim D.-H., Letona A., Lee M., Lim D., Han N.-S., Chung D. (2021). Fluidized-bed granulation of probiotics-encapsulated spray-dried skim milk powder: Effects of a fluidizing aid, moisture-activation and dehydration. Foods.

[2] Fournaise T., Burgain J., Perroud C., Scher J., Gaiani C., Petit J. (2020). Impact of formulation on reconstitution and flowability of spray-dried milk powders. Powder Technol..

[3] Fournaise T., Burgain J., Perroud-Thomassin C., Petit J. (2021). Impact of the whey protein/casein ratio on the reconstitution and flow properties of spray-dried dairy protein powders. Powder Technol..

[4] Barbosa-Cánovas G.V., Ortega-Rivas E., Juliano P., Yan H. (2006). Food Powders: Physical Properties, Processing, and Functionality.

[5] Parikh D.M. (2005). Handbook of Pharmaceutical Granulation Technology.

[6] Shanmugam S. (2015). Granulation techniques and technologies: Recent progresses. BioImpacts.

[7] Pietsch W. (2003). An interdisciplinary approach to size enlargement by agglomeration. Powder Technol..

[8] Pietsch W.B. (2008). Agglomeration Processes: Phenomena, Technologies, Equipment.

[9] Morin G., Briens L. (2014). A comparison of granules produced by high-shear and fluidized-bed granulation methods. Aaps. Pharmscitech..

[10] Badawy S.I., Narang A.S., LaMarche K., Subramanian G., Varia S.A. (2012). Mechanistic basis for the effects of process parameters on quality attributes in high shear wet granulation. Int. J. Pharm..

[11] Bansal A.K., Balwani G., Sheokand S. (2019). Critical material attributes in wet granulation. Handbook of Pharmaceutical Wet Granulation.

[12] Cuq B., Mandato S., Jeantet R., Saleh K., Ruiz T. (2013). Agglomeration/granulation in food powder production. Handbook of Food Powders.

[13] Ullah I., Corrao R., Wiley G., Lipper R. (1987). Moisture activated dry granulation: A general process. Pharm. Technol..

[14] Ullah I., Wang J., Chang S.-Y., Wiley G.J., Jain N.B., Kiang S. (2009). Moisture-activated dry granulation—Part I: A guide to excipient and equipment selection and formulation development. Pharm. Technol..

[15] Ullah I., Wang J., Chang S.-Y., Guo H., Kiang S., Jain N.B. (2009). Moisture-activated dry granulation part II: The effects of formulation ingredients and manufacturing-process variables on granulation quality attributes. Pharm. Technol..

[16] Christensen L., Johansen H., Schaefer T. (1994). Moisture-activated dry granulation in a high shear mixer. Drug Dev. Ind. Pharm..

[17] Moravkar K.K., Ali T.M., Pawar J.N., Amin P.D. (2017). Application of moisture activated dry granulation (MADG) process to develop high dose immediate release (IR) formulations. Adv. Powder Technol..

[18] Chen C.-M., Alli D., Igga M.R., Czeisler J.L. (1990). Comparison of moisture-activated dry granulation process with conventional granulation methods for sematilide hydrochloride tablets. Drug Dev. Ind. Pharm..

[19] Takasaki H., Yonemochi E., Messerschmid R., Ito M., Wada K., Terada K. (2013). Importance of excipient wettability on tablet characteristics prepared by moisture activated dry granulation (MADG). Int. J. Pharm..

[20] Becker D., Rigassi T., Bauer-Brandl A. (1997). Effectiveness of binders in wet granulation: A comparison using model formulations of different tabletability. Drug Dev. Ind. Pharm..

[21] Takasaki H., Yonemochi E., Ito M., Wada K., Terada K. (2015). The importance of binder moisture content in Metformin HCL high-dose formulations prepared by moist aqueous granulation (MAG). Results Pharma Sci..

[22] Astina J., Sapwarobol S. (2019). Resistant maltodextrin and metabolic syndrome: A review. J. Am. Coll. Nutr..

[23] Stępień A., Witczak M., Witczak T. (2020). Sorption properties, glass transition and state diagrams for pumpkin powders containing maltodextrins. LWT-Food Sci. Technol..

[24] Pai D.A., Vangala V.R., Ng J.W., Ng W.K., Tan R.B.H. (2015). Resistant maltodextrin as a shell material for encapsulation of naringin: Production and physicochemical characterization. J. Food Eng..

[25] Bronlund J., Paterson T. (2004). Moisture sorption isotherms for crystalline, amorphous and predominantly crystalline lactose powders. Int. Dairy J..

[26] Terpou A., Papadaki A., Lappa I.K., Kachrimanidou V., Bosnea L.A., Kopsahelis N. (2019). Probiotics in food systems: Significance and emerging strategies towards improved viability and delivery of enhanced beneficial value. Nutrients.

[27] Vesterlund S., Salminen K., Salminen S. (2012). Water activity in dry foods containing live probiotic bacteria should be carefully considered: A case study with *Lactobacillus rhamnosus* GG in flaxseed. Int. J. Food Microbiol..

[28] Chávez B., Ledeboer A. (2007). Drying of probiotics: Optimization of formulation and process to enhance storage survival. Drying Technol..

[29] Neffe-Skocińska K., Rzepkowska A., Szydłowska A., Kołożyn-Krajewska D. (2018). Trends and possibilities of the use of probiotics in food production. Alternative and Replacement Foods.

[30] Bates S., Zografi G., Engers D., Morris K., Crowley K., Newman A. (2006). Analysis of amorphous and nanocrystalline solids from their X-ray diffraction patterns. Pharm. Res..

[31] Atalar I., Yazici F. (2019). Effect of different binders on reconstitution behaviors and physical, structural, and morphological properties of fluidized bed agglomerated yoghurt powder. Drying Technol..

[32] Jinapong N., Suphantharika M., Jamnong P. (2008). Production of instant soymilk powders by ultrafiltration, spray drying and fluidized bed agglomeration. J. Food Eng..

[33] USP (2012). U.P. 35–NF 30. Proceedings of the United States Pharmacopeial Convention.

[34] Sperling L.H. (2006). Introduction to Physical Polymer Science.

[35] Li J., Tao L., Dali M., Buckley D., Gao J., Hubert M. (2011). The effect of the physical states of binders on high-shear wet granulation and granule properties: A mechanistic approach toward understanding high-shear wet granulation process. Part I. Physical characterization of binders. J. Pharm. Sci..

[36] Li J., Tao L., Dali M., Buckley D., Gao J., Hubert M. (2011). The effect of the physical states of binders on high-shear wet granulation and granule properties: A mechanistic approach toward understanding high-shear wet granulation process. Part II. Granulation and granule properties. J. Pharm. Sci..

[37] AOAC (2000). Method 930.15. Official Methods of Analysis of AOAC International.

[38] Yu D., Kwon G., An J., Kim H.J., Chung D. (2021). Glass transition and stickiness characteristics of sea tangle powder fermented with *Lactobacillus brevis*. J. Food Process Preserv..

