# Supplementary material for: High-Shear Granulation of Hygroscopic Probiotic-Encapsulated Skim Milk Powder: Effects of Moisture-Activation and Resistant Maltodextrin
Source: Pharmaceuticals (Basel). 2023 Jan 31;16(2):217. doi: 10.3390/ph16020217 (PMC9962946; doi:10.3390/ph16020217)
Supplement: Supplementary file 1 [file pharmaceuticals-16-00217-s001.zip › pharmaceuticals-2106700-supplementary.pdf]

pharmaceuticals

MDPI

Supporting Materials

High-Shear Granulation of Hygroscopic Probiotic-Encapsulated Skim Milk Powder: Effects of Moisture-Activation and Resistant Maltodextrin

Andres Letona [1], Sungahm Ahn [2], Suyeon An [2], Daebeom Yun [2], Young-Rok Kim [3], Mario Muralles [4] and Donghwa Chung [1,2,5,*]

[1] Institute of Food Industrialization, Institutes of Green Bio Science and Technology, Seoul National University, Pyeongchang 25354, Republic of Korea
[2] Food Technology Major, Graduate School of International Agricultural Technology, Seoul National University, Pyeongchang 25354, Republic of Korea
[3] Department of Food Science and Biotechnology, Institute of Life Science and Resources, Kyung Hee University, Yongin 17104, Republic of Korea
[4] School of Materials Science and Engineering, Nanyang Technological University, 50 Nanyang Avenue, Singapore 639798, Singapore
[5] Center for Food and Bioconvergence, Seoul National University, Seoul 08826, Republic of Korea
* Correspondence: dchung@snu.ac.kr (D.C.)

Table S1. Moisture content (M), water activity (a_w) and viable cells (log (CFU g^{-1})) during high-shear granulation process conducted for the production of Control- and RM-granules.

	Control-granules		
	**M (% dry-basis)**	**a_w**	**Log (CFU g^{-1})**
SD powder	5.10 ± 0.06^d	0.11 ± 0.01^d	8.75 ± 0.24^a
Premixture	4.90 ± 0.05^d	0.17 ± 0.00^d	7.33 ± 0.20^b
Agglomerated mixture	8.81 ± 0.02^a	0.54 ± 0.04^a	7.30 ± 0.03^b
Pre-granules	7.53 ± 0.09^b	0.39 ± 0.03^b	7.26 ± 0.04^b
C-Granule	6.86 ± 0.21^c	0.25 ± 0.03^c	6.60 ± 0.34^c
	**RM-granules**		
	**M (% dry-basis)**	**a_w**	**Log (CFU g^{-1})**
SD powder	5.10 ± 0.06^d	0.11 ± 0.01^d	8.75 ± 0.24^a
Premixture	5.07 ± 0.36^d	0.20 ± 0.01^c	7.31 ± 0.09^b
Agglomerated mixture	10.15 ± 0.21^a	0.54 ± 0.01^a	7.24 ± 0.08^b
Pre-granules	7.72 ± 0.50^b	0.47 ± 0.02^b	7.26 ± 0.08^b
RM-Granules	6.00 ± 0.10^c	0.19 ± 0.02^c	7.29 ± 0.09^b

Values with different letters in the same column are statistically different at $p \leq 0.05$ according to Tukey's test.

Table S2. Viable cells (log (CFU g^{-1})) during the storage of Control- and RM-granules at 25 °C for 28 days.

Time (days)	Control-granules
	Log (CFU g^{-1})
0	6.60 ± 0.34[a]
7	6.45 ± 0.07[a]
14	6.37 ± 0.08[ab]
21	6.29 ± 0.06[ab]
28	6.22 ± 0.05[b]
**Time (days)**	**RM-granules**
	**Log (CFU g^{-1})**
0	7.29 ± 0.09[a]
7	7.26 ± 0.08[a]
14	7.22 ± 0.12[a]
21	7.18 ± 0.36[a]
28	7.14 ± 0.41[a]

Values with different letters in the same column are statistically different at $p \leq 0.05$ according to Tukey's test.

Table S3. Volume-weighted mean diameter ($d_{4,3}$) of each ingredient used in high-shear granulation process.

Ingredient	$d_{4,3}$ (μm)
SD powder	14.51 ± 0.05
Lactose monohydrate	110.25 ± 2.16
Microcrystalline cellulose	118.70 ± 0.63
Resistant maltodextrin (RM)	80.67 ± 2.63

Table S4. Classification of powder flowability according to Carr compressibility index (CI) and Hausner ratio (HR) [33].

CI (%)	Flow character	HR
≤ 10	Excellent	1.00–1.11
11–15	Good	1.12–1.18
16–20	Fair	1.19–1.25
21–25	Passable	1.26–1.34
26–31	Poor	1.35–1.45
32–37	Very poor	1.46–1.59
>38	Very, very poor	>1.60

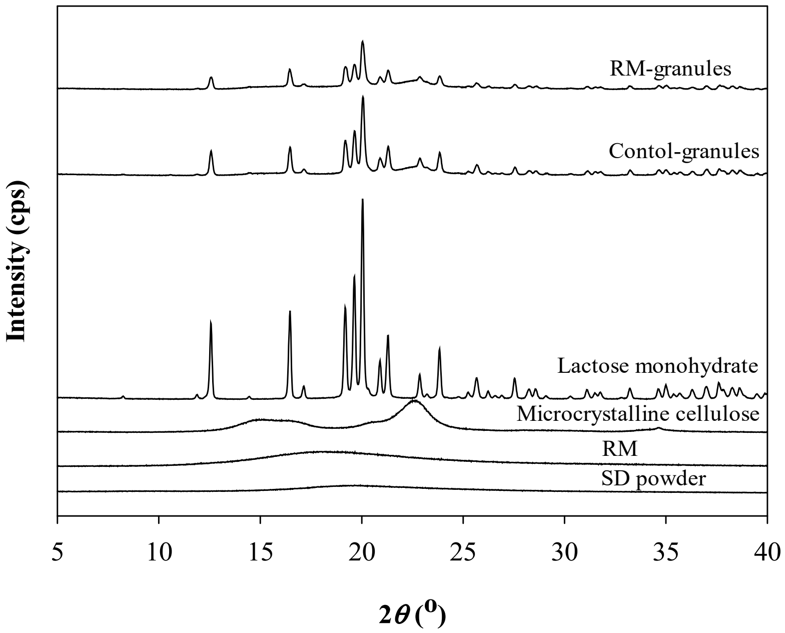

Figure S1. XRD diffractograms of each ingredient used in high-shear granulation process and Control- and RM-granules. The samples were equilibrated at 25 °C and zero RH before analysis.
